# The burden of neural tube defects in Southern Ethiopia: trends, hotspots, and public health implications

**DOI:** 10.7717/peerj.20447

**Published:** 2026-02-17

**Authors:** Beminet Moges Gebremariam, Dejene Hailu Kassa, Barbara J. Stoecker, Afework Mulugeta

**Affiliations:** 1 School of Nutrition, Food Science and Technology, College of Agriculture, Hawassa University, Hawassa, Ethiopia; 2 School of Public Health, College of Medicine and Health Sciences, Hawassa University, Hawassa, Sidama, Ethiopia; 3 Department of Nutritional Sciences, Oklahoma State University, Stillwater, OK, United States of America; 4 Department of Nutrition and Dietetics, School of Public Health, College of Medicine and Health Sciences, Mekelle University, Mekelle, Tigray, Ethiopia

**Keywords:** Neural tube defects, Birth prevalence, Trends, Hotspots, Southern Ethiopia

## Abstract

**Background:**

Neural tube defects (NTDs) are complex multifactorial disorders in the neurulation of the brain and spinal cord that occur between 21 and 28 days after conception. Limited evidence exists regarding the burden of NTDs in Ethiopia, particularly in southern areas. This study aimed to assess the burden, spatial and temporal distribution, and public health implications of neural tube defects in southern Ethiopia.

**Methods:**

This multicenter retrospective study used a structured checklist to collect data from records of women who gave birth in the selected hospitals from January 2017 through December 2021. Birth prevalence of NTDs was calculated per 10,000 births, and temporal trends were assessed using the extended Mantel-Haenszel chi-square test. Spatial analysis was performed to identify hotspot areas. Data were analyzed using SPSS version 25 and ArcGIS version 10.1.

**Results:**

A total of 199,353 babies were delivered at the selected hospitals during the study period, and 320 had NTDs. The overall prevalence of NTDs was 16.1 (95% CI [14.34–17.91]) per 10,000 births. Halaba Kulito General Hospital and Worabe Comprehensive Specialized Hospital reported the highest and second-highest prevalence, at 52.1 and 43.3 per 10,000 births, respectively. Anencephaly was the most frequent type (8.6 per 10,000), followed by spina bifida (6.5 per 10,000). Hotspot clusters, ranging from 16.0 to 46.1 per 10,000 births, were identified in the Halaba, Meskan, and Konso districts of southern Ethiopia. Only 36.6% of NTDs were diagnosed by ultrasound during pregnancy. NTDs were more common in females (61.3%) than males, and approximately three-quarters of affected babies were stillborn.

**Conclusion:**

NTDs represent a significant public health concern in southern Ethiopia, with anencephaly and spina bifida being the most prevalent types. No significant linear trend was observed over the five-year period. Most hotspot districts are located within Ethiopia’s Great Rift Valley. Strengthening prenatal NTD screening, establishing a national digital birth and birth defect registry, and promoting periconceptional folic acid supplementation alongside nutrition education are critical for reducing the burden of NTDs in Ethiopia.

## Background

Neural tube defects (NTDs) are complex multifactorial disorders affecting brain and spinal cord neurulation that occur in humans between 21 and 28 days after conception (Pitkin, 2007; Zaganjor et al., 2016). NTDs are categorized into open and closed defects. Open defects include anencephaly, craniorachisis, and myelomeningoceles, whereas closed defects include encephalocele, meningocele, and spina bifida occulta. Spina bifida and anencephaly are the most common neural tube defects (Avagliano et al., 2019; Copp & Greene, 2013).

Every year, an estimated 300,000 newborns worldwide are born with neural tube defects, resulting in 8.6 million disability-adjusted life years (Zaganjor et al., 2016). In low-income countries, NTDs may account for 29% of neonatal deaths due to observable birth defects (Blencowe et al., 2010). A study in Africa revealed the prevalence of NTDs was 50.71 per 10,000 births (Atlaw et al., 2021). Three Ethiopian studies in different regions revealed that the prevalence of NTDs ranged from 28.6, 167.4, and 215 cases per 10,000 births in Bahir Dar, Amhara region (Mekonnen, MollaTaye & Worku, 2021), Jimma (Silesh et al., 2021), and Tigray region (Mekonen et al., 2021), respectively. Ethiopia had a 4–23 times higher adjusted NTD-related death rate (104.0 per 10,000 births [92.9–116.4]) than any other location reported in sub-Saharan Africa and Southeast Asia (Madrid et al., 2023).

Diverse factors are involved in the causation of NTDs, and genetics and environmental factors were the main etiologies (Agopian et al., 2013; Detrait et al., 2005). Genetic factors include simple gene mutation, and chromosomal abnormalities including trisomies 13 and 18 (De Marco et al., 2011b; Finnell et al., 2021). Environmental factors, including maternal folate intake, age, ethnicity, chronic conditions like diabetes and obesity, and vitamin B12 deficiency have been associated with NTDs (Cao, Xie & Zhang, 2022; Imbard, Benoist & Blom, 2013; Molloy et al., 2009). NTDs have been linked to the use of anti-epileptic and commonly used over-the-counter medications during pregnancy, including psychiatric drugs, antibiotics, and nonsteroidal anti-inflammatory drugs (NSAIDs) (Diamanti et al., 2022; Esposito et al., 2021; Hill et al., 2010; Ornoy, 2009).

Children with NTDs generally have minimal to no bladder and/or bowel control, hip, knee, and foot deformities, and anesthesia of skin (Gober & Gater, 2022; Thompson, 2009). Babies with anencephaly die soon after birth (Dickman & Redfern, 2016; Salari et al., 2022), whereas those with spina bifida have significant socioeconomic impacts and adult mortality (Copp et al., 2015; Oakeshott et al., 2010). Patients with NTDs have a high lifetime direct medical cost, with inpatient care, pediatric therapy, and adult comorbidities accounting for the majority of expenses (Grosse et al., 2016; Oakeshott et al., 2010; Yi et al., 2011). The prevalence of NTDs, particularly folic acid-preventable spina bifida and anencephaly can be reduced to 0.5 per 1,000 live births by consuming adequate amounts of folic acid (Kancherla, Pachón & Oakley Jr, 2021).

Women who take adequate folic acid supplements before and during the first trimester of pregnancy can avoid the majority of neural tube defects (De-Regil et al., 2015). The World Health Organization neural tube defect prevention standard states all women, from the moment they begin trying to conceive until 12 weeks of gestation, should take a folic acid supplement (WHO, 2007). Even though periconceptional use of folic acid would be a simple and useful approach, this opportunity is frequently missed (Czeizel et al., 2013). Ethiopia has failed to initiate periconceptional folic acid supplementation in a timely manner, and despite the Ethiopian Standard Counsel’s endorsement of mandated folate fortification of edible oil and wheat flour, statewide folate fortification has not yet begun. Thus, there is a shortage of evidence on the burden of NTDs in Ethiopia, which policymakers and programmers need to develop comprehensive interventions in the country. The study aimed to assess the burden, spatial and temporal distribution, and public health implications of neural tube defects in southern Ethiopia.

## Material and Methods

### Study setting

The study was conducted at selected hospitals in the Sidama Regional State (SRS) and former South Nation Nationality Peoples Regional States (SNNPRS), which currently include three regions: the Southwestern Peoples Region (SWPR), Central Ethiopian Region (CER), and South Ethiopia Regions (SER). These regions were estimated to account for 20% (20 million) of Ethiopia’s population in 2019 (CSA, 2019). Eleven public hospitals were chosen from a total of 17 public hospitals with general or higher levels, as well as one primary hospital to represent lower levels of hospitals. We included two hospitals from SRS, two from SWPR, four from CER, and four from SER.

### Study design and period

A multicenter retrospective study was conducted at selected hospitals in southern Ethiopia using records for five-year period, from January 2017 to December 2021. This study was carried out between April 14, 2022, and March 27, 2023.

### Population of the study

The study population included all women who delivered their babies at the selected hospitals between January 2017 and December 2021. Cases of neural tube defects included anencephaly, spina bifida, and encephalocele, which can occur alone or in combination with other congenital anomalies categorized by the International Classification of Diseases Clinical Modification Codes, tenth edition (ICD-10) (WHO/CDC/ICBDSR, 2020). Spina bifida occulta was not found in the women’s documentation; hence some occulta diagnoses may have been included in the “spina bifida, unspecified” category.

### Participant selection

Participants’ records were from women who gave birth at 1 primary, 6 general, 4 comprehensive specialized, and 1 teaching hospital across four regions. The study included all babies diagnosed with neural tube defects by an authorized health professional between January 1, 2017, and December 30, 2021, as documented in delivery registration logbooks. The present study included both live births and stillbirths. We were unable to include 36 cases due to the absence of a maternal chart, insufficient documentation, or a record that lacked more than 50% of the recorded values according to the exclusion criteria (Fig. S1).

### Data collection technique and quality assurance

A structured checklist was used to collect data after reviewing various literature. The checklist included sociodemographic, maternal reproductive history, obstetric history, neonatal birth outcomes, morbidity, drug use, and post-birth folic acid supplement intake characteristics. The data collection tool was pretested and modified based on the findings. Data was collected by 12 midwifery professionals with BSc degrees and three supervisors with MSc degrees in health science. Trained data collectors carefully examined delivery registration books and medical charts of women who gave birth at the respective hospitals. NTDs were diagnosed by health professionals and confirmed through documentation of the status and profession of the health workers who diagnosed them during antenatal care, ultrasound reports on women’s medical charts, and delivery registration books.

We gathered spatial data at the district level using mothers’ residences. Data was acquired from medical records and delivery registration books with hospital administration’s permission. The Institutional Review Board of Hawassa University (IRB), Ethiopia, approved this study under the IRB 025/14. Because we retrieved data from medical records, the IRB waived the requirement for informed consent from study participants for the current investigation. To maintain privacy and confidentiality, all information was kept anonymous and adhered to the ethical code for human subjects based on the Helsinki Declaration (World Medical Association, 2013).

### Operational definitions

**Burden (birth prevalence) of NTDs** was calculated by dividing the birth of neonates with neural tube defect cases (LBs and SBs) by total live births and stillbirths within the given period of time per 10,000.

**Multiple NTDs** was defined by a simultaneous occurrence of more than one NTD in a single case.

### Data management and analysis procedures

All collected data was checked manually for completeness and consistency. After cleaning, the acquired data was coded, entered into the EPI INFO version 7.2.0.1 software and transferred to the Statistical Package for Social Science (SPSS) version 25 (IBM Corp., Armonk, NY, USA) software for further analysis. Descriptive statistics were used to describe socio-demographic data, pregnancy care, obstetric history, time to NTD diagnosis, and related congenital anomalies, as well as to estimate the birth prevalence of individuals with neural tube defects. The linear trend of NTD was determined over five years, and the linear trend of the Extended Mantel-Haenszel chi-square was performed. The hot spot analysis was done using ArcGIS version 10.1 software. The hotspot mapping was computed using the collected latitude and longitude of each woman’s residence district and study hospital and recorded in a CSV file. The shapefile for study districts and hospitals was created using row point data, and the point feature was linked to the base map layer of the regions that included study districts and hospitals for women with NTD cases.

## Results

### Sociodemographic characteristics

In the present study, a total of 199,353 live and stillborn babies were delivered at the selected hospitals, and 320 of these babies developed NTDs during the period from 2017 to 2021. Halaba Kulito General Hospital had the highest number of NTD cases, 70 (21.9%). Nearly two-thirds, 206 (64.4%), of the mothers were from the rural areas. The mean age of the women in this study was 26.43 (±5.3 SD), with 23 (7.2%) adolescent women and 181 (56.5%) aged 25-34 years. Among the babies born, 196 (61.3%) were female, with a male-to-female ratio of 1:1.6. (Table 1).

**Table 1:** Socio-demographic characteristics of participants with NTD infants: data from 2017–2021 in southern Ethiopia, 2023 (*n* = 320).

Variables	Frequency	Percent
**Study hospitals**		
Hawassa University Comprehensive Specialized Hospital	25	7.8
Yirgalem General Hospital	2	0.6
Halaba Kulito General Hospital	70	21.9
Butajira General Hospital	68	21.3
Worabe Comprehensive Specialized Hospital	30	9.4
Mizan-Tepi University Teaching Hospital	27	8.4
Gebretsdik Shawo Memorial General Hospital	8	2.5
Jinka General Hospital	23	7.2
Karat Primary Hospital	15	4.7
Arbaminch General Hospital	27	8.4
Wolita Sodo University Comprehensive Specialized Hospital	16	5.0
Wachemo University Nigest Eleni Mohammed Memorial Comprehensive Specialized Hospital	9	2.8
**Residence**		
Rural	206	64.4
Urban	114	35.6
Mean maternal age (years)	26.43 ± 5.3	
**Maternal Age (years)**		
17–19	23	7.2
20–24	85	26.6
25–34	181	56.5
≥35	31	9.7
**Sex of the newborn**		
Male	124	38.8
Female	196	61. 2

### Reproductive history and pregnancy care service

More than half of the women (58.4%) had 2–4 pregnancies during their reproductive journey. Fifteen percent of women had an abortion during a previous pregnancy; four had more than one abortion. Over two-thirds of women (71.6%) obtained antenatal care. During the current pregnancy, 194 (60.6%) women received abdominal ultrasound examinations, with public health facilities providing a large percentage of these services (83.0%). About half (49.1%) of women who gave birth to babies with NTDs were referred by other public hospitals (Table 2).

**Table 2:** Reproductive history and pregnancy care services of women: data from 2017–2021 in southern Ethiopia, 2023 (*n* = 320).

Variables	Frequency	Percent
**Gravidity**		
Once	80	25.0
2–4 times	187	58.4
5 and more times	53	16.6
**Abortion history**		
Yes	48	15. 0
No	272	85.0
**Abortion frequency (*n* = 48)**		
Once	44	91.7
Twice	4	8. 3
**ANC service use**		
Yes	229	71.6
No	91	28.4
**Maternal care delivery**		
Care within the hospital	67	20.9
Referred from public health center	85	26.6
Referred from other public hospital	157	49.1
Referred from private health facilities	11	3.4
**Abdominal ultrasound examination service use**		
Yes	194	60.6
No	126	39.4
**Place for abdominal ultrasound examination** (*n* = 194)		
Public health facility	161	83.0
Private health facility	18	9.3
Both public and private health facility	15	7.7

### Obstetric history and birth outcomes

More than a quarter (26.9%) of women who delivered babies with NTDs encountered complications during pregnancy, the most frequent of which were polyhydramnios (15.9%) and antepartum hemorrhage (2.8%). Most women (87.8%) had spontaneous vaginal deliveries. Almost all women were stable after delivery but two (0.6%) died after childbirth. The majority of babies (98.1%) were singletons, with more than half (54.7%) born at full term. Stillbirths accounted for three-quarters (76.2%) of all birth outcomes, with 94 (38.5%) being macerated. More than half (56.3%) of the babies had low birth weight, and 23 (30.3%) were in respiratory distress (APGAR score < 7 at 5 min). Most living neonates with NTDs (89.5%) were admitted to the NICU (Table 3).

**Table 3:** Obstetric history and birth outcomes of women: data from 2017–2021 in southern Ethiopia, 2023 (*n* = 320).

Variables	Frequency	Percent
**Obstetric complication**		
Pre-eclampsia and Eclampsia	8	2.5
APH	9	2.8
PPH	3	0.9
PROM	5	1.6
Polyhydramnios	51	15.9
Other	10	3.1
None	234	73. 2
**Mode of delivery**		
Spontaneous vaginal	281	87.8
Caesarean section	26	8.1
Forceps/vacuum	6	1.9
Episiotomy	7	2.2
**Maternal health status**		
Stable	317	99.1
Deteriorated or referred	1	0.3
Died	2	0.6
**Newborn delivery**		
Single	314	98.1
Twins	6	1.9
**Neonatal age at birth**		
Preterm	114	35.6
Term	175	54.7
Post-term	2	0.6
Not documented	29	9.1
**Newborn birth weight in gm**		
Low birth weight (Less than 2,500)	180	56.3
Normal weight (2,500 to 4,000)	133	41.6
Over weight (Above 4,000)	3	0.9
Not documented	4	1.3
**Birth outcome**		
Alive	76	23.8
Stillbirth	244	76.2
**Type of stillbirth (*n* = 244)**		
Macerated	94	38.5
Fresh	86	35.3
Not documented	64	26.2
**Respiratory distress (APGAR 5th min)** (*n* = 76)		
Respiratory distress (<7)	23	30.3
Normal neonate (7–10)	53	69.7
**Intervention for alive newborn** (*n* = 76)		
Transferred to NICU	68	89.5
Not documented	8	10.5

**Notes.**

APGAR Appearance Pulse Grimace Activity and Respiration APH Antepartum Hemorrhage NICU Neonatal Intensive Care Unit PPH Postpartum Hemorrhage PROM Premature Rupture of Membranes

### Morbidity, drug use and post birth folic acid supplement Prescription

During pregnancy, 11 women (34%) had reported morbidity, with hepatitis B infection (0.9%) and malaria (0.9%) being the most common. Only seven women (2.2%) used drugs while pregnant. After the delivery of a newborn with NTDs, a quarter (27.2%) of the mothers received a prescription of folic acid supplements during the postnatal period, while two-thirds (67.8%) of the women received a prescription of folic acid-only tablets (Table 4).

**Table 4:** Morbidity, drug use, and post-birth folic acid prescribed for women: data from 2017–2021 in southern Ethiopia, 2023 (*n* = 320).

Variables	Frequency	Percent
**Morbidity during pregnancy**		
Yes	11	3.4
No	309	96.6
**Types of morbidity before or during pregnancy**		
Hepatitis B infection	3	0.9
Malaria	3	0.9
Pyelonephritis	1	0.3
Severe Anemia	2	0.6
Diabetes mellitus	1	0.3
Hypertension	1	0.3
Not documented	309	96.6
**Any drug used during pregnancy**		
Yes	7	2.2
No	313	97.8
**Types of drugs use during pregnancy**		
Ceftriaxone	1	0.3
Coartem	3	0.3
Cobalamin and folic acid	1	0.3
Metformin+	1	0.3
MgSO4 + Hydralazine	1	0.3
Not documented	313	97.8
**Folic acid ordered for women post NTDs birth**		
Yes	87	27.2
No	233	72.8
**Types of folic acid supplement ordered at post NTDs birth** (*n* = 87)		
Iron-folic acid tablet	28	32.2
Folic acid only tablet	59	67.8

### Neural tube defect diagnosis and associated congenital anomalies

Nearly two-thirds (63.4%) of NTDs were diagnosed after the baby was born, while one-third (36.6%) were detected before birth using abdominal ultrasound. Approximately 41% of NTDs were detected with ultrasound between 28.0 to 36.9 weeks of gestation. Only seven (2.2% of NTDs) were recurrent cases. Congenital anomalies accounted for nearly a quarter (23.7%) of neural tube defect cases, with hydrocephalus (81.6%) being the most frequent, followed by clubfoot (11.8%). Midwives diagnosed NTDs at a rate of 55.3%, alongside medical doctors (GPs) at 17.8% and IESO health workers at 11.3% (Table 5).

**Table 5:** Neural tube defect diagnosis and associated congenital anomalies among deliveries: data from 2017–2021 in southern Ethiopia, 2023 (*n* = 320).

Variables	Frequency	Percent
**Time for NTDs diagnosis**		
During pregnancy	108	33.8
After arrival to facility and before delivery care	9	2.8
After the baby delivered	203	63.4
**NTDs diagnosed by ultrasound during pregnancy**		
Yes	117	36.6
No	203	63.4
**Gestational age for first diagnosis with U/S (Weeks) (*n* = 117)**		
≤20	3	2.6
21.0–27.9	33	28.2
28.0–36.9	48	41.0
37.0–42.0	15	12.8
Not documented	18	15.4
**Recurrence of NTD cases**		
Yes	7	2.2
No	313	97.8
**Congenital anomalies associated with NTDs**		
Yes	76	23.7
No	244	76.3
**Types of congenital anomalies associated with NTDs (*n* = 76)**		
Hydrocephalus	62	81.6
Club foot	9	11.8
Abdominal anomalies	3	4.0
Mixed hydrocephaly and club foot	2	2.6
**Health professionals diagnosed NTDs**		
Gynecologist	17	5.3
Medical Doctor (GP)	57	17.8
Midwifery	177	55.3
IESO	36	11.3
Radiologist	10	3.1
More than one professional	23	7.2

**Notes.**

IESO Integrated Emergency Surgery Officers GP General Practitioner

### Birth prevalence of neural tube defects

Out of 199,353 babies (live and stillbirths) delivered during the study period, 320 (124 male and 196 female) had NTDs. The birth prevalence of NTDs was 16.1 (95% CI [14.34–17.91]) per 10,000 births. Halaba Kulito General Hospital (GH) and Worabe Comprehensive Specialized Hospital (CSH) had the highest and second-highest prevalence of NTDs among babies, at 52.1 and 43.3 per 10,000 births, respectively. Yirgalem GH had the lowest NTDs, 1.4 per 10,000 births (Table 6).

**Table 6:** Birth prevalence of neural tube defects among newborn babies: data from 2017–2021 in southern Ethiopia, 2023.

Regions	Hospitals	5 Years			Total NTDs	NTDs per 10,000 births	95% CI per 10,000	
		LB	SB	TB			Lower	Upper
Sidama	Hawassa University CSH	18,752	1097	19,873	25	12.6	8.1	18.6
	Yirgalem GH	14,004	623	14,323	2	1.4	0.2	5.0
	Sum	32756	1720	34,196	27	7.9	5.2	11.5
Central Ethiopia	Halaba Kulito GH	12,980	559	13,444	70	52.1	40.6	65.4
	Butajira GH	20,304	410	20,765	68	32.7	25.4	41.5
	Worabe CSH	6,696	237	6933	30	43.3	29.2	61.7
	Wachemo University NEMMCSH	32,992	930	33,931	9	2.7	1.2	5.0
	Sum	72,972	2136	75,073	177	23.6	20.2	27.3
South western Peoples	Mizan-Tepi University TH	14,304	668	14,814	27	18.2	12.0	26.5
	Gebretsdik Shawo Memorial GH	11,199	482	11,185	8	7.2	3.1	14.1
	Sum	25,503	1150	25,999	35	13.5	9.4	18.7
South Ethiopia	Jinka GH	12,758	571	13,325	23	17.3	11.0	25.9
	Karat PH	9183	198	9436	15	15.9	8.9	26.2
	Arbaminch GH	16,014	510	16,524	27	16.3	10.8	23.8
	Wolita Sodo University CSH	24,208	622	24,800	16	6.5	3.7	10.5
	sum	62163	1901	64,085	81	12.6	10.0	15.7
	Total	193394	6907	199,353	320	16.1	14.3	17.9

**Notes.**

CSH Comprehensive Specialized Hospital GH General Hospital PH Primary Hospital TH Teaching Hospital

### Types of neural tube defects

**Figure 1:**
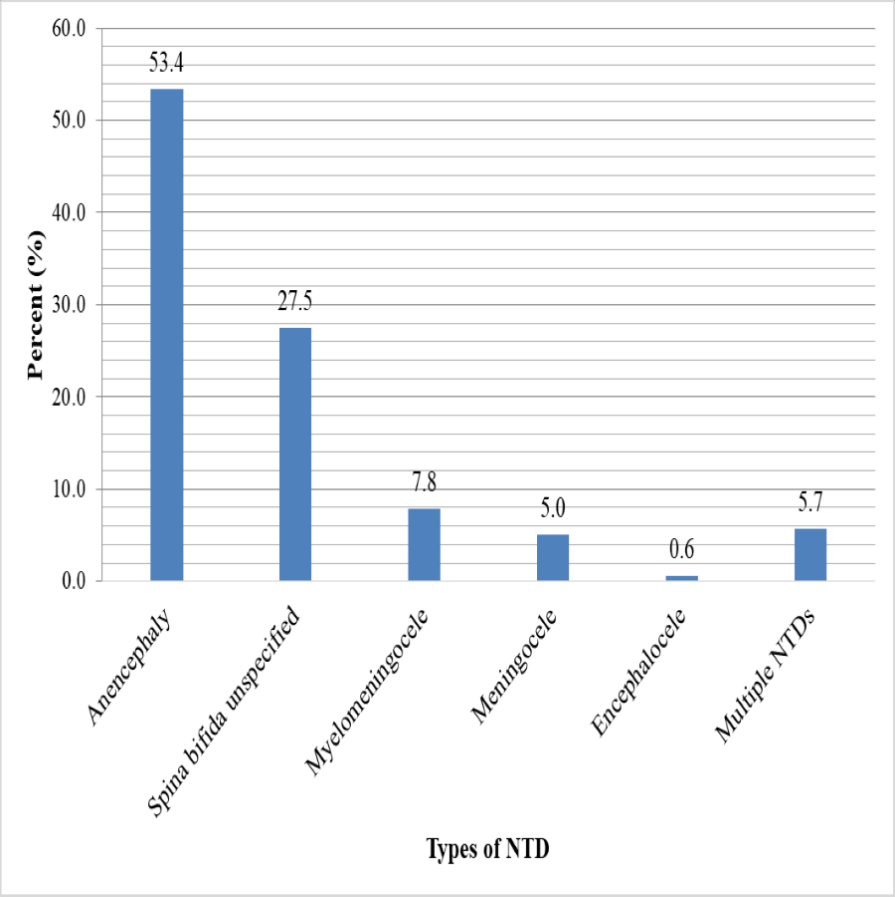
Types of neural tube defects among newborn babies from 2017–2021 at selected hospitals in southern Ethiopia.

In the current study, anencephaly had the highest birth prevalence at 8.6 (95% CI [7.34–9.96]) per 10,000 births, followed by spina bifida combined at 6.5 (95% CI [5.40–7.69]) per 10,000 births. Multiple NTD prevalence was 0.9 (95% CI [0.54–1.43]) per 10,000 births. However, encephalocele had the lowest birth prevalence rate, at 0.1 (95% CI [0.01–0.36]) per 10,000 births (Table 7). Anencephaly accounts for half (53.4%) of all NTD cases, as well as 40.3% of spina bifida, with subtypes spina bifida unspecified, myelomeningocele, and meningocele accounting for 27.5%, 7.8%, and 5%, respectively. While 5.7% of all NTDs had multiple NTDs, only two (0.6%) had encephaloceles (Fig. 1).

**Table 7:** Types of neural tube defects among newborn babies: data from 2017–2021 in southern Ethiopia, 2023 (*n* = 320).

Type of neural tube defects	*n*	(%)	Total birth prevalence per 10,000 births	95% CI Per 10,000	
				Lower	Upper
Anencephaly	171	53.4	8.6	7.34	9.96
Spina bifida	129	40.3	6.5	5.40	7.69
Encephalocele	2	0.6	0.1	0.01	0.36
Multiple NTDs	18	5.7	0.9	0.54	1.43
Total	320		16.1	14.34	17.91

### Birth outcomes of neural tube defects

**Figure 2:**
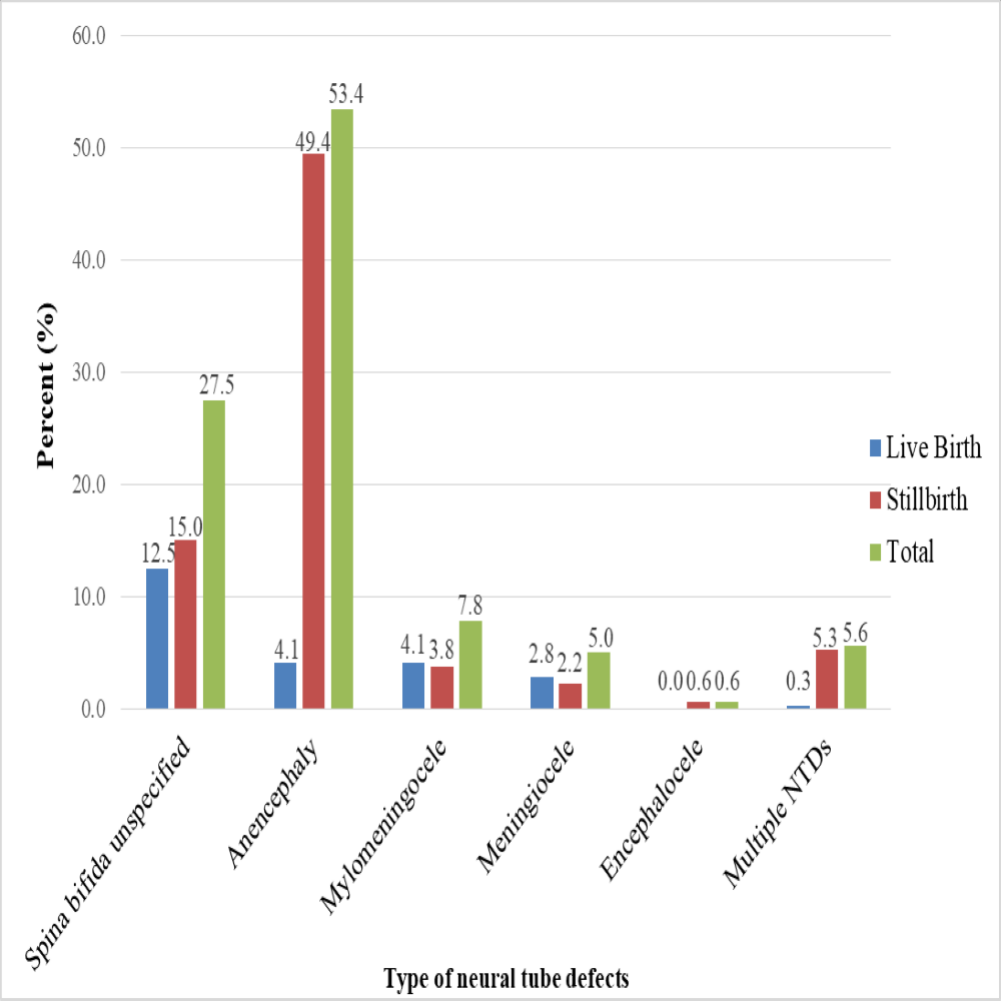
Types of neural tube defects and birth outcomes data from 2017–2021 at selected hospitals in southern Ethiopia.

In this study, stillbirths accounted for three-quarters (76.2%) of all NTD deliveries. Macerated stillbirths accounted for 38.5% of all stillbirths. Among the types of NTDs, about half (49.4%) of anencephaly cases were stillbirths, with only 4.1% being live births, whereas 21.0% of all spina bifida cases (spina bifida unspecified, myelomeningocele, and meningocele) were stillbirths, and 19.4% of spina bifida cases were born alive. All cases of encephalocele (0.6%) resulted in stillbirths (Fig. 2).

### Trends of neural tube defects in southern Ethiopia

**Figure 3:**
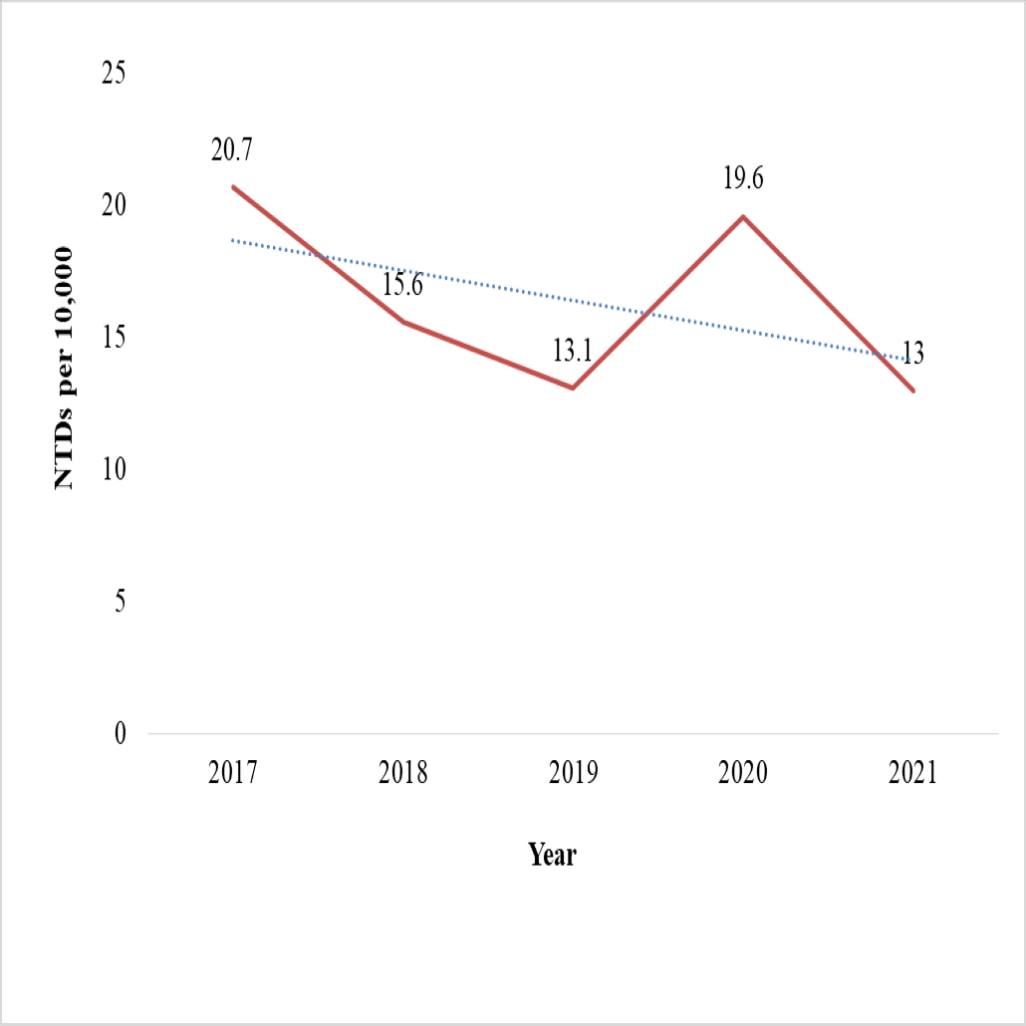
Five-year trends of neural tube defects from 2017–2021 at selected hospitals in southern Ethiopia.

**Figure 4:**
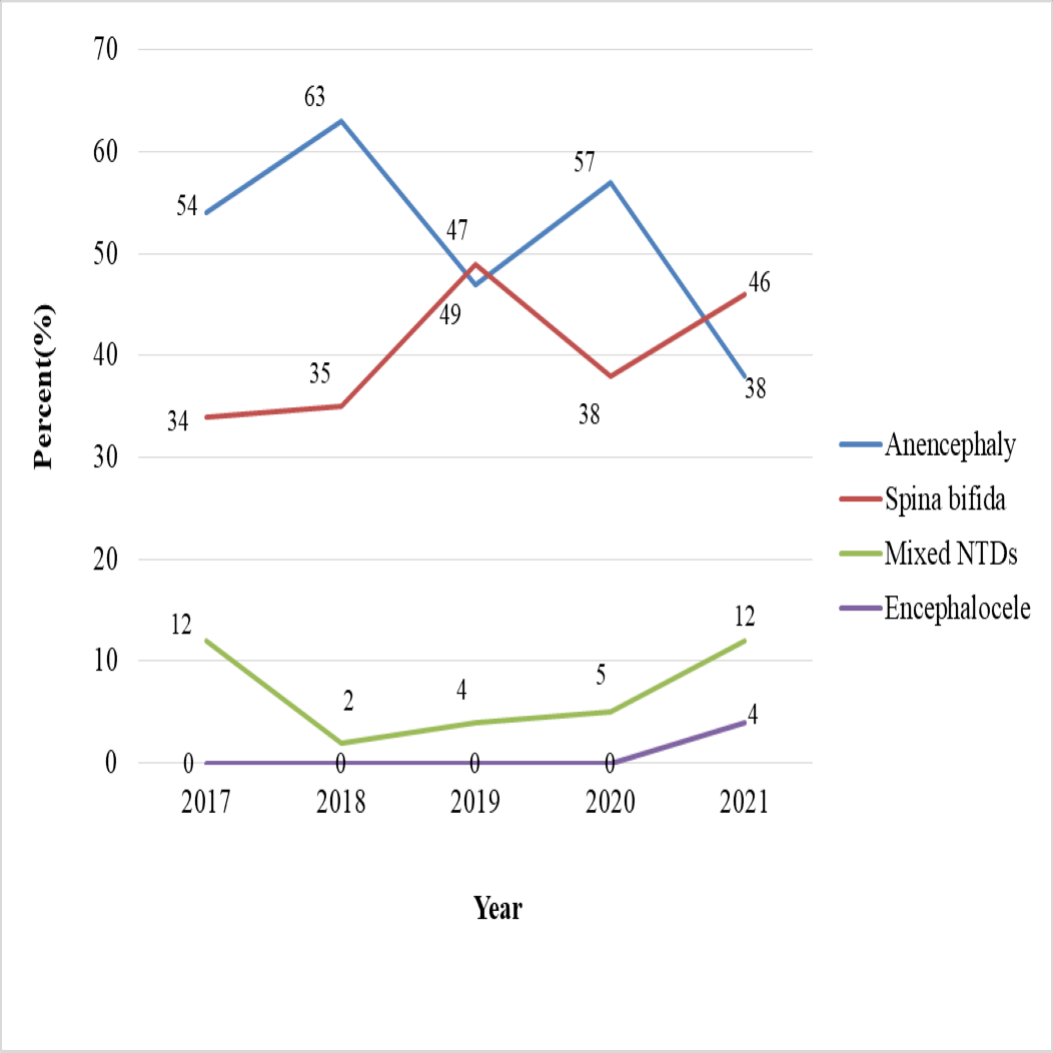
Trends of neural tube defect types from 2017 to 2021 at selected hospitals in southern Ethiopia.

The chi-square test for linear trend (Extended Mantel-Haenszel) did not show a statistically significant relationship between the proportion of NTD cases and the five-year period (*χ*^2^ = 2.7, *p* = 0.09). Odds ratios have decreased in some years, most notably in 2018 (OR = 0.75), 2019 (OR = 0.63), and 2021 (OR = 0.63) (Table 8). The birth prevalence of NTDs showed a general decline over the five-year period, dropping from 20.7 in 2017 to 13.0 per 10,000 births in 2021. A notable slope occurred in 2019 (13.1 per 10,000 births), followed by a transient increase in 2020 (19.6 per 10,000). While anencephaly cases decreased, the highest was 63% in 2018 and the lowest was 38% in 2021. However, the rate of spina bifida cases has risen from 34% in 2017 to 46% in 2021 (Figs. 3 and 4).

**Table 8:** Linear trends of neural tube defects among newborns: data from 2017–2021 in southern Ethiopia, 2023.

Year	LB	SB	Total No. newborns delivered	No. of newborns with NTDs	NTDs per 10,000 birth	Proportion	Mantel-Haenszel summary odds ratio
2017	29,717	1,295	30,978	64	20.7	0.21	1
2018	35,335	1,340	36,573	57	15.6	0.16	0.75
2019	41,817	1,437	42,900	56	13.1	0.13	0.63
2020	40,054	1,412	41,274	81	19.6	0.2	0.95
2021	46,471	1,423	47,628	62	13.0	0.13	0.63
Total	193,394	6,907	199,353	320	16.1	0.16	

**Notes.**

Extended Mantel-Haenszel chi square for linear trend over time = 2.72 (*P* = 0.098).

LB live birth SB stillbirth

### Spatial hotspot mapping of neural tube defects in southern Ethiopia

**Figure 5:**
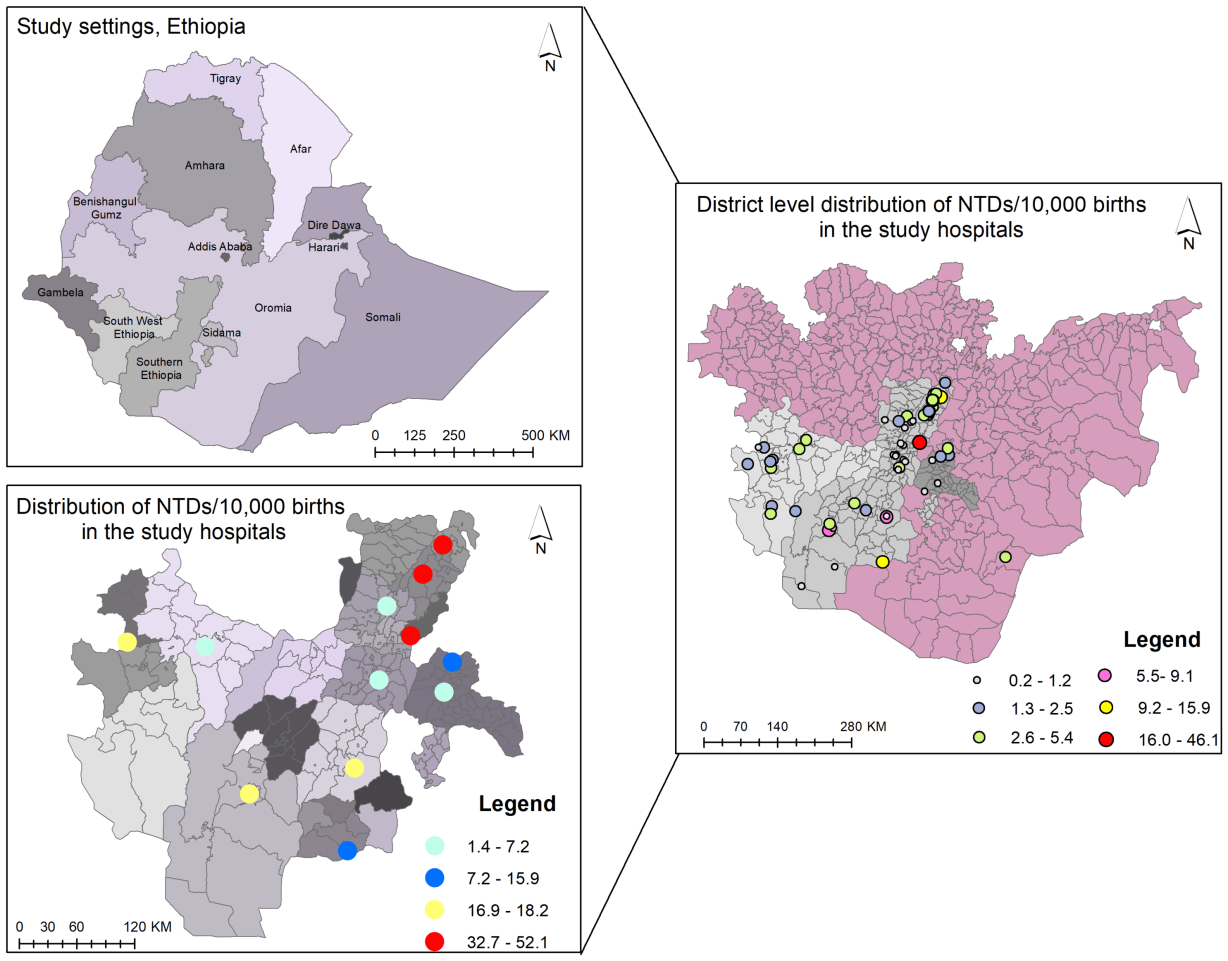
Spatial hotspot mapping of neural tube defects among newborns from 2017–2021 in southern Ethiopia. District-level hotspots per 10,000 births include very high (red spot): 16–46.1; high (orange spot): 10.0–15.9; moderate (yellow spot): 5.6–9.1; mild (purple spot): 2.6–5.4; low (blue spot): 1.3–2.5; and very low (green spot): 0.2–1.2 hotspot districts. Hospital-level hotspots per 10,000 births include very high (red spot): 32.7–52.1; high (light yellow spot): 16.9–18.2; moderate (blue spot): 12.6–15.9; and mild (light blue spot): 1.4–7.2 hotspot hospitals.

In the present study, we performed a spatial hotspot mapping of NTDs. The district-level distribution showed that the Halaba, Meskan, and Konso districts had the highest frequency of NTD cases, with rates ranging from 16.0 to 46.1 per 10,000 births. These NTD hotspots account for 34% of the overall number of NTD cases in Southern Ethiopia. The second hotspot location was the Arbamich, Butajira, and Jinka town districts, where rates ranged from 10.0 to 15.9 per 10,000 births, accounting for 14% of all NTD cases in southern Ethiopia (Fig. 5). The spatial autocorrelation report of the district-level special analysis was a z-score of 0.04 (*p* = 0.96), and the pattern does not appear to be statistically significant (Moran’s Index = 0.0022) (Fig. S2). There is no statistically significant evidence of clustering. However, the majority of the NTD hotspot districts in this study were located in Ethiopia’s Great Rift Valley. A hospital-based hotspot mapping for NTD cases revealed that three hospitals, Butajira GH, Worabe CSH, and Halaba Kulito GH, had the most NTD cases, with caseloads ranging from 32.7 to 52.1 per 10,000 births The spatial autocorrelation has a *z*-score of 0.07 (*p* = 0.9461), and the pattern does not appear to be statistically significant (Moran’s Index = −0.077610) (Fig. S3).

## Discussion

Between 2017 and 2021, 199,353 babies (96.5% live births and 3.5% stillbirths) were born at the selected hospitals. The overall NTD birth prevalence was 16.1 (95% CI [14.34–17.91]) per 10,000 births. The birth prevalence of neural tube defects in this study was consistent with a study in northern Ghana that found 16 NTD cases per 10,000 births (Alhassan, Adam & Nangkuu, 2017). However, the NTD birth prevalence was higher than in an European study, at 9.1 per 10,000 births (Khoshnood et al., 2015), 4.0 per 10,000 births in Nepal (Bhandari et al., 2015), and 7.8 per 10,000 births in Korea (Lamichhane et al., 2016). Additionally, NTD prevalence was 9.9 per 10,000 live births in four hospitals in Dar es Salaam, Tanzania (Kishimba et al., 2015), 10.3 per 10,000 births in Kampala, Uganda (Mumpe-Mwanja et al., 2019), and 11.7 per 10,000 births in eight WHO member countries in Africa (Zaganjor et al., 2016). The discrepancy in NTD birth prevalence rates could be attributed to differences in implementation of nutritional intervention, maternal care, and methodology, as well as socioeconomic inequities.

Our study found a lower birth prevalence of NTDs than previous studies in Liaoning Province, China (19.1 per 10,000 live births) (Zhang et al., 2017), Eritrea (39 per 10,000 births) (Estifanos et al., 2017), Tigray region (Mekonen et al., 2021), Jimma (Silesh et al., 2021), Addis Ababa (Gedefaw, Teklu & Tadesse, 2018), the Amhara region (Abdu & Seyoum, 2019), and Eastern Ethiopia (Berhane & Belachew, 2022), which reported 215, 167.4, 126, 53.5, and 107.5 per 10,000 births, respectively. The low prevalence in this study could be attributed to the inability to include terminated cases due to a lack of NTD diagnosis as a reason for termination, as well as to incomplete data from hospital abortion registration books. Another possible explanation is that the study was conducted in southern Ethiopia, where people may consume more folate-rich fruits and green leafy vegetables through traditional meal preparations such as gomen kitfo (minced kale), gomen beayeb (cheese mixed with kale), kurkufa with kale or aleko (moringa), gomen besega (kale mixed with meat), and other foods.

The birth prevalence of anencephaly in southern Ethiopia was 8.6 (95% CI: 7.34, 9.96) per 10,000 births, based on our findings. In this study, the birth prevalence of anencephaly was higher than that reported in previous studies from Addis Ababa and Amhara (4.7 per 10,000 births) and Northwest Ethiopia (5.1 per 10,000 births) (Seyoum & Adane, 2018; Taye et al., 2019). In contrast, the study’s anencephaly prevalence is less than 56.4 per 10,000 births in North Shewa (Kindie & Mulu, 2022) and 26.9 per 10,000 births in Jimma, Ethiopia (Silesh et al., 2021).

According to this five-year retrospective analysis, the birth prevalence of spina bifida is 6.5 (95% CI [5.40–7.69]) per 10,000 births. This prevalence is consistent with a study in Liaoning Province, China, which found 6.25 per 10,000 births (Zhang et al., 2017). Similarly, the prevalence of spina bifida was lower than reported in Northern Ghana, at 9 per 10,000 births (Alhassan, Adam & Nangkuu, 2017). Although the prevalence of spina bifida in this study was lower than 64.4 in Tigray (Berihu et al., 2018), it was 51.9 per 10,000 babies in Addis Ababa, Ethiopia (Gedefaw, Teklu & Tadesse, 2018), and 13.4 per 10,000 births in the Felege-Hiwot Comprehensive Specialized Referral Hospital in Bahir Dar, Ethiopia (Mekonnen, MollaTaye & Worku, 2021). The variations in spina bifida prevalence can be attributed to a combination of genetic, environmental, regional, ethnic, and racial groups and socioeconomic factors. These factors contributed to differences in maternal nutrition, particularly folic acid intake, exposure to teratogens, genetic predisposition, and variations in healthcare access.

Encephalocele had the lowest birth prevalence rate in our study, at 0.1 per 10,000 births. The reported rate is lower than a study in Nigeria (66.9 per 10,000 births) (Toma et al., 2018), as well as two studies conducted at teaching hospitals in Addis Ababa: 3.5 (Gedefaw, Teklu & Tadesse, 2018) and 1.7 (Sorri & Mesfin, 2015) encephaloceles per 10,000 births. The disparities could be attributed to several factors, including geographical location, genetic predisposition, environmental influences, including deficiencies in folic acid and exposure to toxins or specific dietary habits, and improved access to antenatal care and skills for detecting and reporting the cases.

In regard to hospital-based prevalence in Ethiopia, Halaba Kulito GH and Worabe CSH had the highest and second-highest rates of NTDs in babies, with 52.1 and 43.3 per 10,000 births, respectively. This could be attributable to women’s lack of folic acid supplementation during the periconceptional period, as well as a lack of early antenatal care visits, food preferences, and short birth intervals. The finding, backed by research in California, USA (Todoroff, 2000), revealed a link between short birth intervals and NTDs, related to inadequate maternal recovery and nutritional depletion, particularly folate deficiency. Additionally, a study conducted in Dessie Town demonstrated that khat chewing during pregnancy was linked to the birth of babies with anencephaly (Abebe et al., 2021). Khat use during pregnancy may be linked to obstetric issues like low birth weight (LBW), anemia, hypertension, intrauterine fetal death (IUFD), and embryotoxic effects (Abdel-Aleem, 2015; Dendir & Deyessa, 2017; Kedir, Berhane & Worku, 2013). Nevertheless, women who chew khat without realizing the risks it presents to their unborn child (Hunde et al., 2023).

The most prevalent congenital malformations associated with NTDs were hydrocephalus and clubfoot. The findings were similar to those reported in Sub-Saharan Africa and Tigray, Ethiopia (Berihu et al., 2018; Warf, 2011). Possibly NTDs are caused by abnormal neural plate closure, which can lead to hydrocephalus. In addition, a further reason for links between hydrocephalus and NTDs is the Chiari II malformation, which prevents cerebrospinal fluid (CSF) flow.

The current study found no statistically significant evidence of a linear trend, and there was no consistent increase or decrease in the proportion of NTDs across the five-year period. In contrast, other studies in Eastern Ethiopia and Jimma, Ethiopia, reported a linear increasing trend (Berhane & Belachew, 2022; Silesh et al., 2021). These discrepancies in NTD trends could be attributed to health professionals’ limited competence to diagnose NTDs, inconsistencies in folic acid supplementation and maternal care, and hospitals’ inability to implement a standard national computerized birth and birth defect registration system. The birth prevalence of NTDs declined over a five-year period, from 20.7 per 10,000 births in 2017 to 13.0 per 10,000 births in 2021. A notable slope occurred in 2019 (13.1 per 10,000 births), followed by a transient increase in 2020 (19.6 per 10,000 births). This could be related to pregnant women’s limited access to prenatal care and folic acid supplementation as a result of nationwide lockdowns and disruptions to health services during the COVID-19 pandemic. Despite the fluctuations, the fitted trend line shows a progressive drop over a five-year period. Regarding NTD types, anencephaly declined; the highest percentage of NTD cases (63%) and the lowest percentage (38%) were reported in 2018 and 2021, respectively. Conversely, the percentage of cases of spina bifida rose from 34% in 2017 to 46% in 2021.

A district-level spatial hotspot analysis of neural tube defects showed that the highest incidence of NTDs, ranging from 16.0 to 46.1 per 10,000 births, was among mothers residing in the Halaba, Meskan, and Konso districts of southern Ethiopia. The second hotspot location, with rates ranging from 10.0 to 15.9 per 10,000 births, included women from Arbamich, Butajira, and Jinka towns. Even though, there is no statistically significant evidence of clustering, we observed that the majority of the NTD hotspot districts in this study were located in Ethiopia’s Great Rift Valley. This finding is supported by a study in Kenya that found significant levels of NTDs in the Rift Valley provinces (Githuku et al., 2014). This could be attributed to high temperatures or prolonged exposure to sunlight during storage or transportation of folate foods, as folates are sensitive to heat, atmospheric oxygen, and UV radiation (Delchier et al., 2016; Siatka, Mát’uš & Moravcová, 2025).

Nearly half of babies with neural tube defects (47.8%) were born to mothers aged 26 to 35. These findings are consistent with previous study results conducted at Debre Berhan Comprehensive Specialized Hospital (Mulu et al., 2022) and Hiwot Fana Specialized University Hospital in Harar, Ethiopia (Edris et al., 2020). Most investigations found a link between prior spontaneous abortion and the incidence of NTDs (Gashaw et al., 2021; Hassan, 2021; Tesfay, Aga & Teshome, 2021). In our study, 15% of women who gave birth to babies with NTDs experienced abortions during previous pregnancies. This finding is consistent with studies in Italy, where 17.3% (De Marco et al., 2011a), and in Eastern Ethiopia, 18.8% (Berhane & Belachew, 2022), of women who gave birth to babies with NTDs had abortions in previous pregnancies. This could be due to the remaining trophoblastic material from the previous pregnancy, which may interfere with fetal development in the present pregnancy, resulting in NTDs (Clarke et al., 1975; Tegene, 2023).

Nearly two-thirds (63.4%) of NTDs were detected after birth, with midwives diagnosing more than half of them. Abdominal ultrasound examination services were used by 60.6% of women, and one-third (36.6%) of NTDs were detected using ultrasonography throughout pregnancy and before delivery. In contrast, 63% in Morocco (Forci et al., 2021) and 93% in Addis Ababa were diagnosed during the antenatal period (Aklilu et al., 2023). Inadequate antenatal care for pregnant women, as well as a lack of professional expertise in diagnosing NTDs with ultrasound examination, may contribute to the low rate of NTD detection during pregnancy. Discovering NTDs in the prenatal stage enables couples to make an informed decision about whether to continue with their pregnancy or have it terminated medically.

The study found that babies with NTDs resulted in stillbirths in three-quarters of cases, and of all the stillbirths, 38.5% were macerated. The findings are consistent with research conducted in Sub-Saharan Africa and Southeast Asia, as well as in Tigray, Northern Ethiopia (Berihu et al., 2018; Madrid et al., 2023). Approximately half (49.4%) of anencephaly and 21.0% of all spina bifida cases (spina bifida unspecified, myelomeningocele, and meningocele) were stillbirths, whereas only 4.1% and 19.4% of anencephaly and spina bifida patients were born alive, respectively. The finding is supported by studies that demonstrate babies with anencephaly are often stillborn, but neonates with spina bifida may live for a few days but require special medical care and surgery (Jaruratanasirikul et al., 2014; Salari et al., 2022). This finding is consistent with another study conducted at Debre Berhan Referral Hospital in North Shewa, Ethiopia (Kindie & Mulu, 2022). This could be related to anencephalic newborns, whose brains do not fully develop and can die in utero or shortly after birth (Salari et al., 2022). In contrast, newborns with spina bifida are at a high risk of physical illness (Gober & Gater, 2022) and cognitive impairment (Lindquist, Strinnholm & Peny-Dahlstrand, 2022).

Neural tube defects affected more female babies (61.3%) than male neonates, with a male–female ratio of 1:1.6. This finding is also supported by research from Northwest Nigeria (Nnadi, 2016), Khartoum, Sudan (Omer, Mohammed & Abbasher, 2016), and two teaching hospitals in Addis Ababa (Sorri & Mesfin, 2015). However, contradicting data from research in Northern Ghana (Alhassan, Adam & Nangkuu, 2017), Tigray (Berihu et al., 2018), and the Bale Zone of Ethiopia (Atlaw et al., 2019) show that males are more afflicted by NTDs than females.

Our study found that 56.3% of babies with NTDs had low birth weight (<2500g). The finding is supported by research conducted at St. Louis Medical Center in the United States of America (Norman et al., 2012), Hiwot Fana Specialized University Hospital, Harar (Edris et al., 2020), and Addis Ababa, Ethiopia (Gedefaw, Teklu & Tadesse, 2018). The link between NTDs and low birth weight babies could be attributed to shared risk factors, including maternal factors such as folate deficiency, malnutrition, or inadequate intake of essential nutrients, as well as exposure to certain medications or radiation during pregnancy, and other environmental factors.

During the postnatal period, folic acid supplement orders were recorded on the order sheet of the women’s chart for a quarter (27.2%) of the mothers of babies with NTDs. Folic acid-only pills were ordered for about 67.8% of women. This finding aligned with research done in Addis Ababa (Yesehak et al., 2023). To avoid NTD recurrence, healthcare providers are encouraged to prescribe 5 mg/d folic acid pills for women who gave birth to NTD babies. However, there is no protocol to ascertain women’s consumption of the supplement. Our identification of NTD hotspots over a wide geographic area in southern Ethiopia provides evidence to support coordinated interventions. The current study’s inclusion of a high number of births is another important strength. One limitation is that this study only included women who gave birth in hospitals; as a result, neonates born outside of hospitals would not have been included and the frequencies of NTDs in the region would have been underestimated. Additionally, because the study was designed to collect data from medical records and birth registries, we were unable to include some important information.

## Conclusions

This study highlights that neural tube defects remain a major public health concern in southern Ethiopia, with anencephaly and spina bifida being the most prevalent types. The majority of identified NTD hotspot districts are located within the Great Rift Valley. Although no consistent trend was observed over the five-year study period, the burden of NTDs was found to be three times higher than the global minimum prevalence for folic acid-preventable spina bifida and anencephaly. Strengthening timely screening services and expanding rehabilitation programs for affected newborns are urgently needed. In addition, establishing a national, digitized birth and birth-defect registration system would allow better monitoring of trends and outcomes to inform interventions. Policy makers and stakeholders should prioritize community-level periconceptional folic acid supplementation, promote nutrition education, and encourage the consumption of folate-rich foods, particularly in NTD hotspot areas and across Ethiopia.

## Supplemental Information

10.7717/peerj.20447/supp-1 Supplemental Information 1 Schematic presentation of sampling procedure of the study in southern Ethiopia

10.7717/peerj.20447/supp-2 Supplemental Information 2 Spatial autocorrelation report of neural tube defect in the study districts of southern Ethiopia

10.7717/peerj.20447/supp-3 Supplemental Information 3 Spatial autocorrelation report of neural tube defect in the study hospitals of southern Ethiopia

10.7717/peerj.20447/supp-4 Supplemental Information 4 SPSS raw data for NTD burden study in southern Ethiopia

10.7717/peerj.20447/supp-5 Supplemental Information 5 Raw data for NTDs hospital data

10.7717/peerj.20447/supp-6 Supplemental Information 6 Spacial hot spot raw data of NTDs in southern Ethiopia

10.7717/peerj.20447/supp-7 Supplemental Information 7 Operational definition of terms

## Additional Information and Declarations

### Data Availability

The following information was supplied regarding data availability: The raw measurements are available in the Supplementary Files.
