# Supplementary figures and images for: The burden of neural tube defects in Southern Ethiopia: trends, hotspots, and public health implications
Source: PeerJ. 2026 Feb 17;14:e20447. doi: 10.7717/peerj.20447 (PMC12922585; doi:10.7717/peerj.20447)

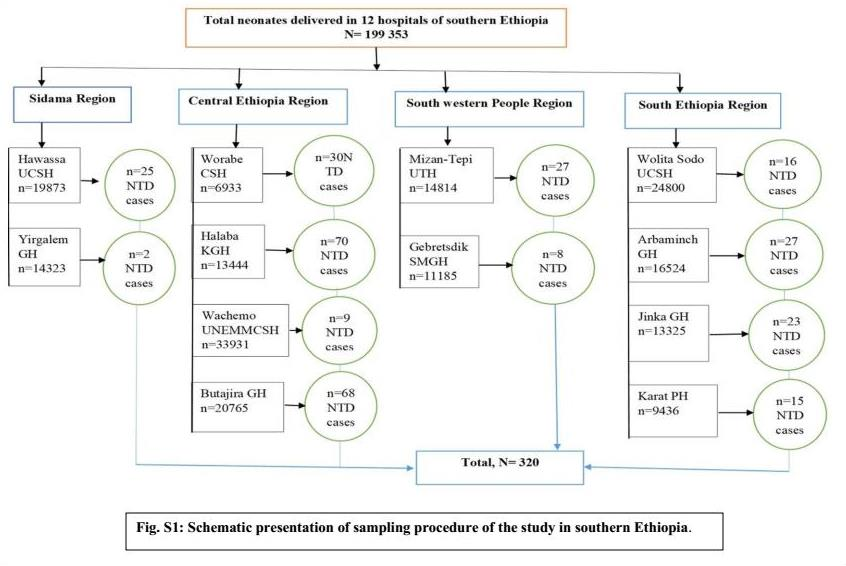

Supplement: Supplemental Information 1 [file peerj-14-20447-s001.tiff]

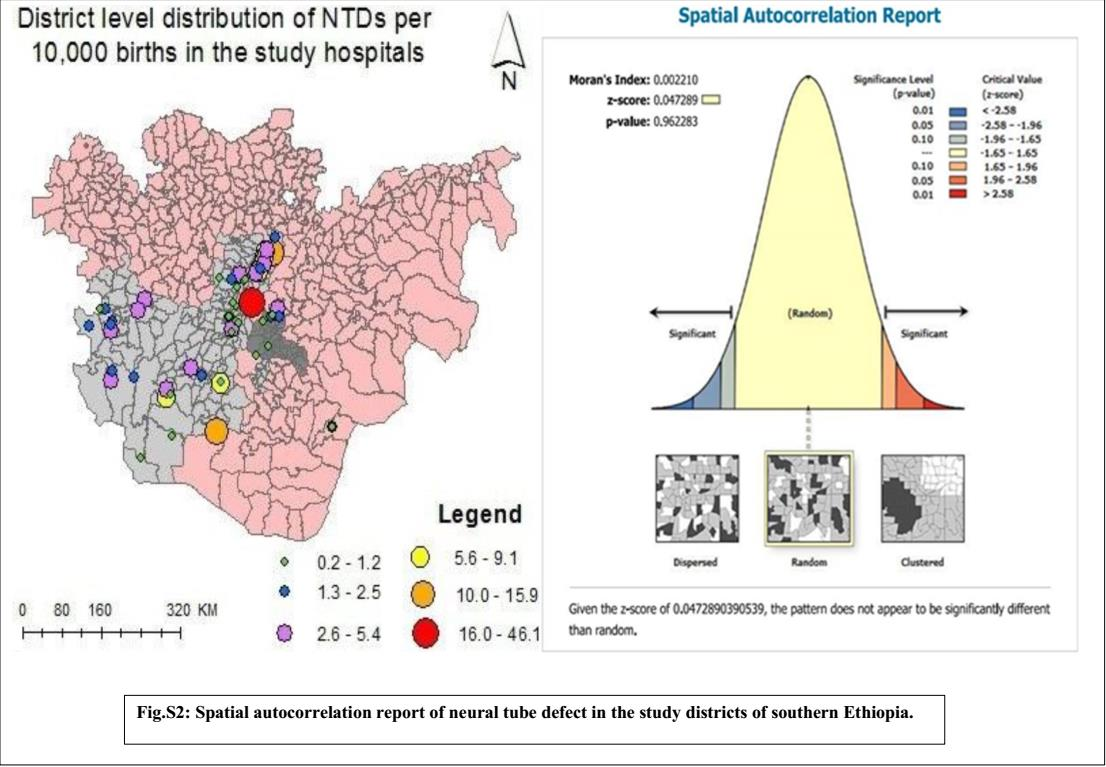

Supplement: Supplemental Information 2 [file peerj-14-20447-s002.tiff]

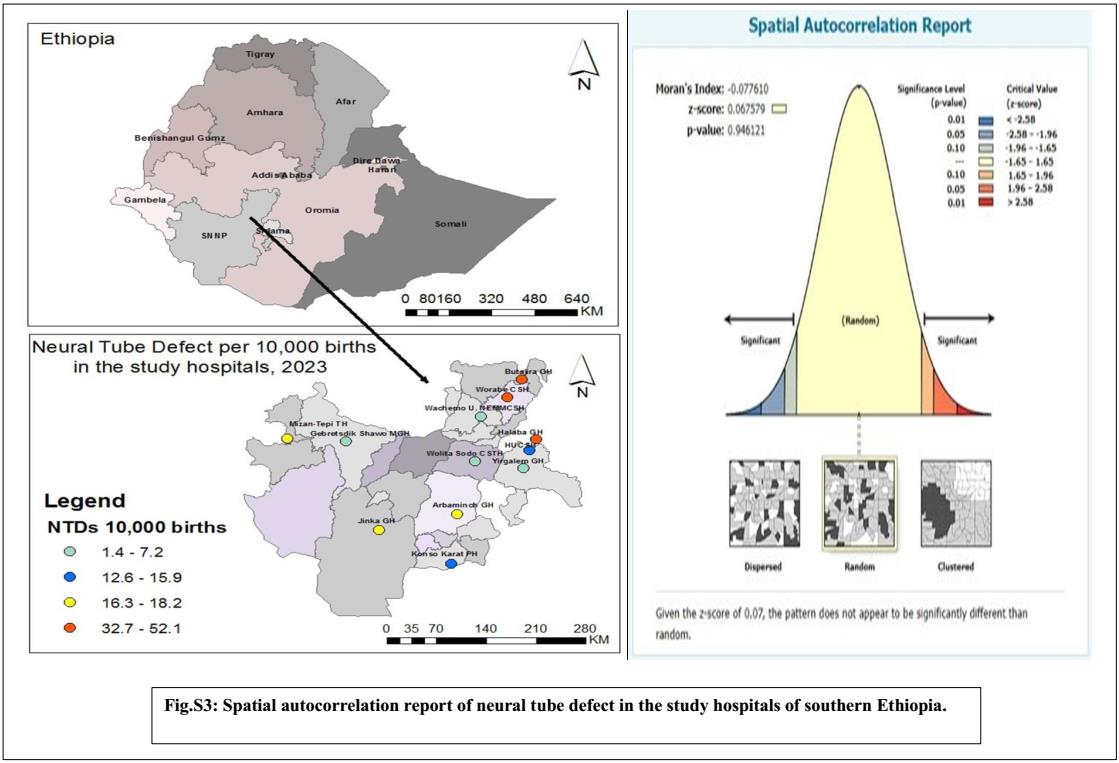

Supplement: Supplemental Information 3 [file peerj-14-20447-s003.tiff]
