# Supplementary material for: The burden of neural tube defects in Southern Ethiopia: trends, hotspots, and public health implications
Source: PeerJ. 2026 Feb 17;14:e20447. doi: 10.7717/peerj.20447 (PMC12922585; doi:10.7717/peerj.20447)
Supplement: Supplemental Information 7 [file peerj-14-20447-s007.pdf]

Operational Definition of Terms

Urban are generally defined as localities with 2000 or more inhabitants based on the Ethiopian Central Statistical Agency.

Rural comprise all areas not classified as urban.

Antenatal care (ANC) is a health service provided to pregnant women in the continuum of maternity care.

ANC service use is the utilization of antenatal care services for at least 1 or more scheduled contacts.

Maternal care delivery is the care delivered to women who attended pregnancy care within the hospital or are referred from public or private health facilities.

Maternal health status is the health and well-being of women during childbirth; it could be stable, dead, deteriorated, or referred.

Neonatal age at birth is the gestational age of the newborn during birth, including premature, full-term, and post-term newborns.

Time for NTDs diagnosed is the time at which the neural tube defects were detected, which includes during pregnancy follow-up, after arrival to the facility and before delivery care, or after the baby was delivered.

Stillbirth is a fetal death occurring before birth, after a selected, predefined duration of gestation. The death of the fetus could have occurred before the onset of labor (antepartum) or at the time of delivery (intrapartum).
